# Celestial polarization patterns sufficient for Viking navigation with the naked eye: detectability of Haidinger's brushes on the sky versus meteorological conditions

**DOI:** 10.1098/rsos.160688

**Published:** 2017-02-08

**Authors:** Gábor Horváth, Péter Takács, Balázs Kretzer, Szilvia Szilasi, Dénes Száz, Alexandra Farkas, András Barta

**Affiliations:** 1 Environmental Optics Laboratory, Department of Biological Physics, Eötvös University, Pázmány sétány 1, 1117 Budapest, Hungary; 2 Danube Research Institute, MTA Centre for Ecological Research, 1113 Budapest, Karolina út 29-31, Hungary; 3 Estrato Research and Development Ltd., Németvölgyi út 91/c, 1124 Budapest, Hungary

**Keywords:** Viking navigation, Haidinger's brushes, sunstone, sky polarization, imaging polarimetry, human polarization vision

## Abstract

If a human looks at the clear blue sky from which light with high enough degree of polarization *d* originates, an 8-shaped bowtie-like figure, the yellow Haidinger's brush can be perceived, the long axis of which points towards the sun. A band of high *d* arcs across the sky at 90° from the sun. A person can pick two points on that band, observe the yellow brushes and triangulate the position of the sun based on the orientation of the two observed brushes. This method has been suggested to have been used on the open sea by Viking navigators to determine the position of the invisible sun occluded by cloud or fog. Furthermore, Haidinger's brushes can also be used to locate the sun when it is below the horizon or occluded by objects on the horizon. To determine the position of the sun using the celestial polarization pattern, the *d* of the portion of the sky used must be greater than the viewer's degree of polarization threshold d* for perception of Haidinger's brushes. We studied under which sky conditions the prerequisite *d* > d* is satisfied. Using full-sky imaging polarimetry, we measured the *d*-pattern of skylight in the blue (450 nm) spectral range for 1296 different meteorological conditions with different solar elevation angles *θ* and per cent cloud cover *ρ*. From the measured *d*-patterns of a given sky we determined the proportion *P* of the sky for which *d* > d*. We obtained that *P* is the largest at low solar elevations *θ *≈ 0° and under totally or nearly clear skies with cloud coverage *ρ* = 0%, when the sun's position is already easily determined. If the sun is below the horizon (−5° ≤ *θ* < 0°) during twilight, *P* = 76.17 ± 4.18% for $d_{\min}^{*}=23\%$ under clear sky conditions. Consequently, the sky-polarimetric Viking navigation based on Haidinger's brushes is most useful after sunset and prior to sunrise, when the sun is not visible and large sky regions are bright, clear and polarized enough for perception of Haidinger's brushes.

## 1. Introduction

**Figure 1.**
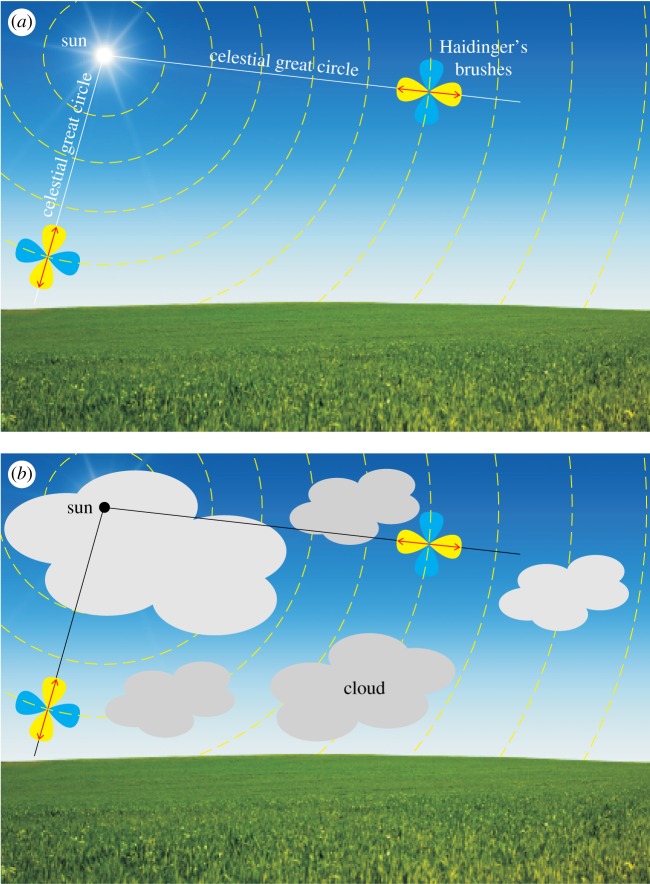
Principle of the sky-polarimetric Viking navigation on the basis of Haidinger's brushes. (*a*) Clear sky. (*b*) Cloudy sky. Yellow bars: local direction of polarization of skylight. Red double-headed arrow: axis of mirror symmetry of the yellow Haidinger's brush being perpendicular to the direction of polarization. The long axis of the blue Haidinger's brush is parallel to the direction of polarization. Owing to Rayleigh scattering of sunlight in the atmosphere, the celestial great circle passing through the observed sky point parallel to the yellow Haidinger's brush crosses the sun.

If the human eye is stimulated by linearly polarized light, two 8-shaped bowtie-like figures are perceived [1,2]: (i) blue brushes parallel to the direction of polarization of the stimulus and (ii) yellow brushes perpendicular to the direction of polarization (figure 1*a*). These two 8-shaped figures combine to form a Maltese cross shape. These are the so-called Haidinger's brushes, the angular extension of which is about 5° centred at the fovea of the human retina, and are mediated by dichroic carotenoids in the macula lutea [1–7]. If somebody looks at the clear blue sky from which light with high enough degree of polarization *d* originates (for example, at 90° from the sun, where *d* is maximal and can reach 75% [2,8]), the yellow Haidinger's brushes can be perceived, while the blue brushes are practically invisible, because they fade into the blue sky (figure 1*a*). The blue brushes could be perceived only if the sky is not blue, i.e. around sunset and sunrise when large portions of the sky are red and orange, or when the sky is covered by grey or white clouds or fog. However, in the latter cases the *d* of skylight can be so low [8–11] that it falls below the perception threshold d* of Haidinger's brushes. The brushes have the best contrast when *d* approaches 100%, for instance looking at a white area on a liquid crystal computer monitor, which employs a linear polarizer as part of the image-forming technology [2,12].

Recently, Temple *et al*. [13] measured the degree of polarization threshold d* at which humans could just detect Haidinger's brushes. They presented gratings in polarization-only contrast at varying degrees of polarization *d* in order to measure the lower limits of human polarized light detection. Participants were, on average, able to perform the task down to a threshold of $d_{\mathrm{ave}}^{*}=56\pm 3\%$, and there was a normal continuous distribution of threshold values extending down to as low as $d_{\min}^{*}=23\%$ and up to as high as $d_{\max}^{*}=87\%$ [13].

Owing to the first-order Rayleigh scattering, at first approximation, the direction of polarization of skylight is perpendicular to the scattering plane determined by the relative position of the observer, the sun and the celestial point observed (figure 1). Using full-sky imaging polarimetry, Suhai & Horváth [14] and Hegedüs *et al*. [9,10] showed that depending on the cloud conditions, a considerable part of the sky is characterized by directions of polarization described well by the Rayleigh model. Moreover, those sky areas where the Rayleigh model can be considered as a good approximation of the real direction of skylight polarization have the highest *d-*values. Thus, if a human looks at the sky and perceives the yellow Haidinger's brush, the celestial great circle passing through the observed sky point parallel to this yellow brush crosses the sun (figure 1). Consequently, if two sky points are selected with high enough *d*-values to perceive Haidinger's brushes, the sun position can be determined, which is at the intersection of these two great circles (figure 1).

Ropars *et al*. [15,16] suggested that, during their voyages on the open sea, Viking navigators might have been able to determine the position of the invisible sun occluded by cloud or fog in this way (figure 1*b*). The determination of the position of the occluded sun is an essential part of the famous sky-polarimetric Viking navigation hypothesized, cited and disputed by many researchers [10,11,14–38].

The navigation method with Haidinger's brushes is a sound idea and has the advantage that Viking navigators did not need any additional optical instrument (e.g. calcite, cordierite or tourmaline sunstones) to analyse the sky polarization, because due to the perceived yellow Haidinger's brush they had their own analyser embedded in their eyes (figure 1). The weakness of this hypothesis is that the Haidinger's brushes are very faint and fade quickly within a few seconds due to visual adaptation. But they can be perceived again for some seconds if the fovea of the eye is restimulated by polarized skylight after winking or moving or rotating the position or orientation of gaze in the sky.

The atmospheric optical prerequisite of this Viking navigation hypothesis based on the observation of Haidinger's brushes [15,16] is that the degree of polarization *d* of skylight stimulating the human eye should be higher than the perception threshold d* of Haidinger's brushes. The aim of this work was to study under which sky conditions the prerequisite *d* > d* is satisfied. Using full-sky imaging polarimetry, we measured the *d*-pattern of skylight in the blue (450 nm) spectral range for 1296 different meteorological conditions with different solar elevation angles *θ* and per cent cloud cover *ρ* in Hungary (at latitude 47°15′29.83′′ N). From the measured *d*-patterns of a given sky we determined the proportion *P* of the sky for which *d* > d*, that is for which the Haidinger's brushes can be perceived. The entoptic phenomenon of Haidinger's brushes is mediated by the absorption of short-wavelength light by the macular carotenoids (lutein, zeaxanthin and meso-zeaxanthin) and is not visible at wavelengths above approximately 500 nm [39]. Therefore, our study was restricted to the blue colour band because only this is useful for perception of Haidinger's brushes.

## 2. Material and methods

### 2.1. Full-sky imaging polarimetry and selection of different skies

The patterns of the degree of polarization *d* of skylight were measured by imaging polarimetry, the method of which was described in detail by Barta *et al*. [24]. Data on sky polarization have been collected with an automatic full-sky imaging polarimeter set-up in the Gothard Astronomical Observatory of the Eötvös University, Szombathely, Hungary (47°15′29.83′′ N, 16°36′15.67′′ E). In the past 3 years this polarimeter functioned continuously and measured several tens of thousands of sky polarization patterns. We grouped these skies on the basis of the following two parameters: (i) the solar elevation angle *θ* above the horizon ranged from −5° to +55°. This range of *θ* was divided into 12 equal intervals with an increment of 5° as follows: −5° ≤ *θ*_1_ < 0°, 0° ≤ *θ*_2_ < 5°, 5° ≤ *θ*_3_ < 10°, 10° ≤ *θ*_4_ < 15°, 15° ≤ *θ*_5_ < 20°, 20° ≤ *θ*_6_ < 25°, 25° ≤ *θ*_7_ < 30°, 30° ≤ *θ*_8_ < 35°, 35° ≤ *θ*_9_ < 40°, 40° ≤ *θ*_10_ < 45°, 45° ≤ *θ*_11_ < 50°, 50° ≤ *θ*_12_ ≤ 55°. (ii) The per cent cloud cover *ρ* (% of the full sky covered by clouds) was determined with the use of the cloud detection algorithm *k*NN (*k*-nearest neighbour) described by Barta *et al*. [40]. The range 0% ≤ *ρ* ≤ 100% was divided into nine categories, as in meteorology oktas (or eighths) are common units of cloud coverage of the visible sky estimated by visual examination [41]. Oktas 0–8 refer to ever-increasing cloud coverage based on the division of eight equal intervals [42]: 0 okta refers to the totally clear sky (*ρ*_0_ = 0%); there is a category for few clouds (1–2 oktas), another for scattered clouds (3–4 oktas), for broken clouds (5–7 oktas) and the last one for an overcast sky (8 oktas). Based on this generally accepted method, our first category corresponds to the totally clear sky with *ρ*_0_ = 0%, and the further categories are composed of eight equal intervals with an increment of 12.5% in the following way: 0% < *ρ*_1_ < 12.5%, 12.5% ≤ *ρ*_2_ < 25%, 25% ≤ *ρ*_3_ < 37.5%, 37.5% ≤ *ρ*_4_ < 50%, 50% ≤ *ρ*_5_ < 62.5%, 62.5% ≤ *ρ*_6_ < 75%, 75% ≤ *ρ*_7_ < 87.5%, 87.5% ≤ *ρ*_8_ ≤ 100% (nearly and totally overcast sky). For example, if the cloud coverage is 3 oktas, this means that approximately 3/8 part of the visible sky is covered by clouds (in our measurement, it falls into the range of 25% ≤ *ρ*_3_ < 37.5%). It is not worth separating the totally overcast (*ρ* = 100%) case, because in our measurement all the sky conditions in the 8 okta range (87.5% ≤ *ρ*_8_ ≤ 100%) seemed totally overcast, which could not be separated visually. The cloudless case (*ρ*_0_ = 0%) is easy to recognize, therefore, its separation is logical in our measurements.

Thus, we created 12 × 9 = 108 (*θ*, *ρ*) groups. In each group, we selected 12 different sky samples (with *θ*- and *ρ*-values falling into the *θ*- and *ρ*-interval of the given group) from our polarimetric sky archives. Finally, we obtained 108 × 12 = 1296 different sky situations differing in *θ* and *ρ*, but in a given group their *θ*- and *ρ*-values were similar. Further on we used the *d*-patterns of these skies measured by imaging polarimetry in the blue (450 nm) spectral range.

### 2.2. Proportion of the sky with degrees of polarization higher than the perception threshold of Haidinger's brushes

Using the *d*-pattern of a given sky measured in the blue spectral range, we calculated the proportion *P* of the sky for which $d>d_{\min}^{*}=23\%$, $d>d_{\mathrm{ave}}^{*}=56\%$ and $d>d_{\max}^{*}=87\%$ as these d* values were measured by Temple *et al*. [13]. The resulting values of $P_{\min}(d>d_{\min}^{*})$, $P_{\mathrm{ave}}(d>d_{\mathrm{ave}}^{*})$ and $P_{\max}(d>d_{\max}^{*})$ give the probability that a viewer (Viking navigator) with high, average and low polarization sensitivity, respectively, finds celestial points where Haidinger's brushes can be perceived by the naked eye and used for sky-polarimetric Viking navigation. These *P*_min_-, *P*_ave_- and *P*_max_-values were calculated and visualized for the investigated 1296 different sky situations. In the 180° field-of-view circular images of the full sky, the under- and overexposed points as well as landmarks (vegetation, building parts) on the horizon were not taken into consideration. These sky points were masked out during calculations.

## 3. Results

**Figure 2.**
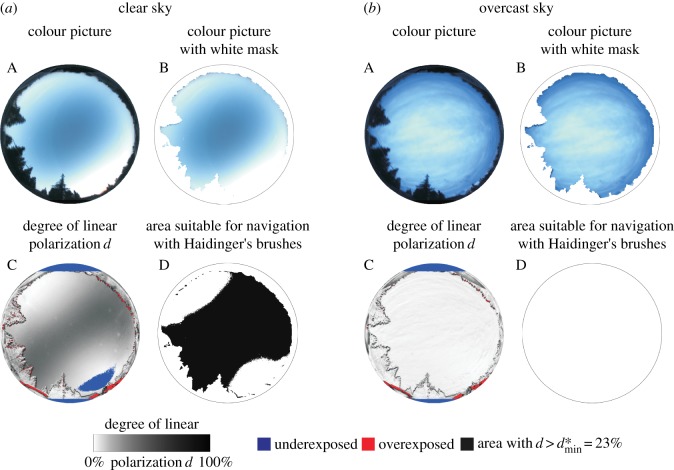
(*a*)(A) A clear sky photographed on 7 June 2014 at 18.36.29 (=GMT + 2 h, where GMT means Greenwich Mean Time) with solar elevation *θ* = 0° and per cent cloud cover *ρ* = 0%. (B) Same as A where the white areas are under- and/or overexposed regions as well as landmarks on the horizon, which are not taken into consideration. The white sky regions were masked out during calculations. (C) Pattern of the degree of linear polarization *d* of skylight measured with full-sky imaging polarimetry in the blue (450 nm) spectral range. (D) Points of the clear sky are marked with black where $d>d_{\min}^{*}=23\%$. (*b*) Same as on the left for a cloudy sky photographed on 11 January 2015 at 15.48.59 (=GMT + 1 h) with solar elevation *θ* = 0° and per cent cloud cover *ρ* = 100%. (D) The circular area is blank, because there is no celestial point with *d* > 23%.

**Figure 3.**
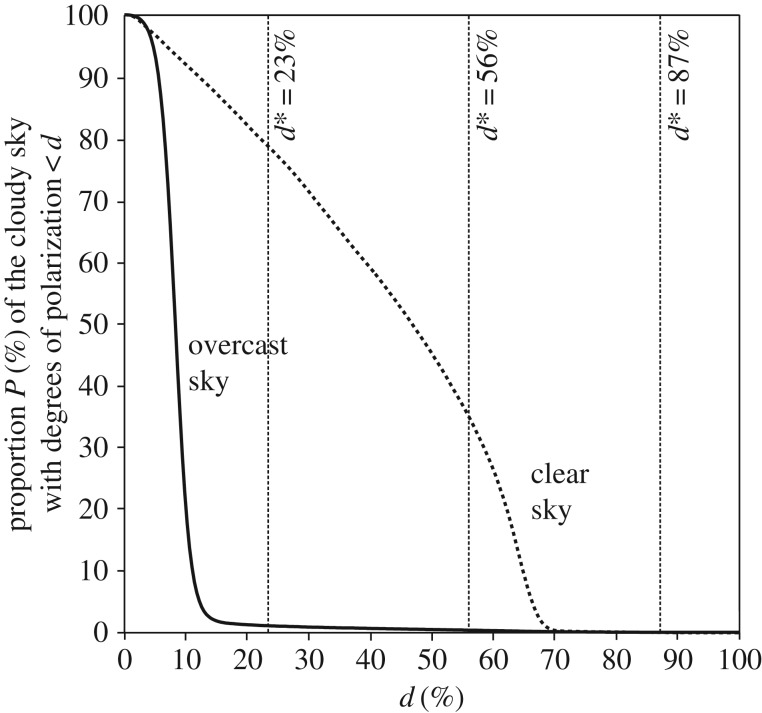
Proportion *P* (%) of the clear and overcast skies in figure 2 with degrees of skylight polarization not lower than *d* in the blue (450 nm) spectral range. The vertical lines show the values d* = 23, 56, 87% being the three characteristic perception thresholds of Haidinger's brushes.

When skies are clear (figure 2), the proportion of the celestial hemisphere *P* suitable for human polarimetric navigation based on perception of Haidinger's brushes is high (figure 3): *P*(d*=23%) = 78%, *P*(d* = 56%) = 35%, *P*(d* = 87%) = 0%. Thus, the most sensitive Viking navigators with 23% ≤ d* ≤ 56% could have perceived Haidinger's brushes in the clear sky of figure 2. However, when the sky is overcast (figure 2), *P* approaches 0%, making it infeasible for navigation by means of this (figure 3).

**Figure 4.**
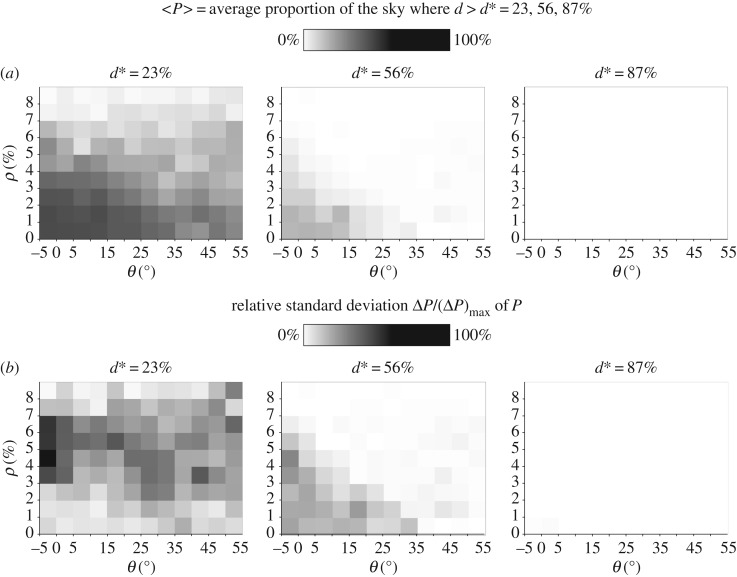
(*a*) The <*P*>(*θ*, *ρ*) matrices calculated for 1296 meteorological situations (characterized by sun elevation *θ* and per cent cloud cover *ρ*) in the blue (450 nm) spectral range for the perception thresholds of Haidinger's brushes $d_{\min}^{*}=23\%$, $d_{\mathrm{ave}}^{*}=56\%$ and $d_{\max}^{*}=87\%$. In every cell of a given matrix there are 12 different skies. Each cell contains the average <*P*> of *P*, where *P* is the proportion of the sky for which *d* > d* (=23, 56, 87%). (*b*) As (*a*) for the Δ*P*/(Δ*P*)_max_(*θ*, *ρ*) matrices, each cell of which contains the relative (normalized) standard deviation Δ*P*/(Δ*P*)_max_ of *P*.

We calculated the 12 × 9-size sun elevation *θ*–cloud cover *ρ* matrices of the average proportion of the sky usable for navigation with Haidinger's brushes for the selected 1296 meteorological situations in the blue (450 nm) spectral range for the perception thresholds of Haidinger's brushes $d_{\min}^{*}=23\%,$ $d_{\mathrm{ave}}^{*}=56\%$ and $d_{\max}^{*}=87\%$ (figure 4*a*). In every cell of a given matrix there are 12 different skies. Each cell contains the average <*P*> of *P*, where *P* is the proportion of the sky for which *d* > d* (= 23, 56, 87%). <*P*> measures the appropriateness of Viking navigation with Haidinger's brushes under a meteorological situation belonging to a given cell (*ρ*, *θ*). In figure 4*b*, each cell of a given matrix contains the relative (or normalized) standard deviation Δ*P*/(Δ*P*)_max_ of *P*. Tables 1–3 and 4–6 contain the numerical data visualized by grey shades in the matrices of figure 4*a* and *b*, respectively.

**Table 1.** Numerical values (%) of the <*P*>(*θ*, *ρ*) matrix in the blue (450 nm) spectral range for $d_{\min}^{*}=23\%$ shown in figure 4*a*. The 12 intervals of solar elevation *θ* are: −5° ≤ *θ*_1_ < 0°, 0° ≤ *θ*_2_ < 5°, 5° ≤ *θ*_3_ < 10°, 10° ≤ *θ*_4_ < 15°, 15° ≤ *θ*_5_ < 20°, 20° ≤ *θ*_6_ < 25°, 25° ≤ *θ*_7_ < 30°, 30° ≤ *θ*_8_ < 35°, 35° ≤ *θ*_9_ < 40°, 40° ≤ *θ*_10_ < 45°, 45° ≤ *θ*_11_ < 50°, 50° ≤ *θ*_12_ ≤ 55°. The nine intervals of cloud per cent *ρ* are: *ρ*_0_ = 0% (clear sky), 0% < *ρ*_1_ < 12.5%, 12.5% ≤ *ρ*_2_ < 25%, 25% ≤ *ρ*_3_ < 37.5%, 37.5% ≤ *ρ*_4_ < 50%, 50% ≤ *ρ*_5_ < 62.5%, 62.5% ≤ *ρ*_6_ < 75%, 75% ≤ *ρ*_7_ < 87.5%, 87.5% ≤ *ρ*_8_ ≤ 100% (nearly and totally overcast sky).

	*θ*_1_ = −5–0°	*θ*_2_ = 0–5°	*θ*_3_ = 5–10°	*θ*_4_ = 10–15°	*θ*_5_ = 15–20°	*θ*_6_ = 20–25°	*θ*_7_ = 25–30°	*θ*_8_ = 30–35°	*θ*_9_ = 35–40°	*θ*_10_ = 40–45°	*θ*_11_ = 45–50°	*θ*_12_ = 50–55°
*ρ*_8_	1.16	4.53	1.46	1.97	4.43	1.36	2.51	2.54	4.99	2.04	7.14	8.11
*ρ*_7_	3.37	4.91	4.10	1.72	9.95	10.93	8.09	10.23	11.73	9.73	13.54	4.29
*ρ*_6_	26.44	16.76	12.64	15.90	10.48	14.48	18.03	8.82	12.78	20.68	21.18	30.47
*ρ*_5_	45.24	29.65	9.95	27.76	30.51	16.91	23.65	23.92	19.16	26.48	26.10	29.93
*ρ*_4_	39.87	34.17	48.33	42.58	31.78	31.56	30.61	34.07	16.21	20.20	30.90	29.32
*ρ*_3_	62.32	59.97	59.23	56.16	48.26	42.36	36.07	22.97	30.15	34.93	29.14	24.48
*ρ*_2_	72.20	72.05	64.12	69.28	59.37	53.03	43.55	33.01	40.63	45.95	34.79	33.59
*ρ*_1_	76.99	74.76	72.55	75.80	69.12	67.98	59.98	52.76	49.94	57.47	51.95	44.57
*ρ*_0_	76.17	75.80	75.37	74.46	69.52	69.37	63.30	59.61	45.51	41.20	54.71	46.98

**Table 2.** Numerical values (%) of the <*P*>(*θ*, *ρ*) matrix in the blue (450 nm) spectral range for $d_{\mathrm{ave}}^{*}=56\%$ shown in figure 4*a*.

	*θ*_1_ = −5–0°	*θ*_2_ = 0–5°	*θ*_3_ = 5–10°	*θ*_4_ = 10–15°	*θ*_5_ = 15–20°	*θ*_6_ = 20–25°	*θ*_7_ = 25–30°	*θ*_8_ = 30–35°	*θ*_9_ = 35–40°	*θ*_10_ = 40–45°	*θ*_11_ = 45–50°	*θ*_12_ = 50–55°
*ρ*_8_	0.31	0.39	0.27	0.29	0.32	0.21	0.29	0.30	0.25	0.20	0.36	0.27
*ρ*_7_	0.30	0.31	0.29	0.27	0.35	0.32	0.26	0.33	0.31	0.29	0.28	0.21
*ρ*_6_	0.89	0.53	0.34	0.48	0.29	0.36	0.35	0.34	0.36	0.55	0.59	0.56
*ρ*_5_	4.60	1.21	0.31	0.28	0.38	0.25	0.38	0.42	0.53	0.74	0.93	0.63
*ρ*_4_	8.77	2.04	1.41	0.78	0.25	0.49	0.58	0.66	0.31	0.57	0.76	0.81
*ρ*_3_	12.76	7.14	2.97	1.52	0.44	0.47	0.41	0.48	0.35	1.31	0.65	0.58
*ρ*_2_	16.72	16.73	6.46	4.82	3.22	1.48	0.40	0.46	0.41	0.77	0.56	0.50
*ρ*_1_	27.79	23.64	15.39	26.65	10.48	6.93	3.10	0.73	0.67	3.05	1.44	0.69
*ρ*_0_	26.40	28.14	26.51	22.85	11.19	6.99	2.40	5.08	0.29	0.21	0.74	0.30

**Table 3.** Numerical values (%) of the <*P*>(*θ*, *ρ*) matrix in the blue (450 nm) spectral range for $d_{\max}^{*}=87\%$ shown in figure 4*a*.

	*θ*_1_ = −5–0°	*θ*_2_ = 0–5°	*θ*_3_ = 5–10°	*θ*_4_ = 10–15°	*θ*_5_ = 15–20°	*θ*_6_ = 20–25°	*θ*_7_ = 25–30°	*θ*_8_ = 30–35°	*θ*_9_ = 35–40°	*θ*_10_ = 40–45°	*θ*_11_ = 45–50°	*θ*_12_ = 50–55°
*ρ*_8_	0.0047	0.0169	0.0051	0.0044	0.0070	0.0010	0.0053	0.0089	0.0044	0.0014	0.0098	0.0031
*ρ*_7_	0.0079	0.0070	0.0051	0.0031	0.0110	0.0128	0.0067	0.0119	0.0118	0.0052	0.0069	0.0011
*ρ*_6_	0.0160	0.0178	0.0168	0.0101	0.0111	0.0139	0.0182	0.0103	0.0105	0.0376	0.0413	0.0164
*ρ*_5_	0.0158	0.0130	0.0084	0.0103	0.0071	0.0112	0.0169	0.0197	0.0217	0.0454	0.0642	0.0370
*ρ*_4_	0.0235	0.0131	0.0160	0.0128	0.0037	0.0224	0.0230	0.0249	0.0097	0.0255	0.0640	0.0629
*ρ*_3_	0.0288	0.0288	0.0196	0.0232	0.0206	0.0141	0.0210	0.0083	0.0134	0.0579	0.0317	0.0365
*ρ*_2_	0.0354	0.0360	0.0385	0.0172	0.0710	0.0105	0.0128	0.0097	0.0029	0.0402	0.0296	0.0191
*ρ*_1_	0.0848	0.0451	0.0450	0.0189	0.0683	0.0417	0.0216	0.0162	0.0013	0.0297	0.0197	0.0328
*ρ*_0_	0.1052	0.1375	0.0653	0.0397	0.0125	0.0109	0.0268	0.0377	0.0038	0.0018	0.0551	0.0082

**Table 4.** Numerical values (%) of the Δ*P*(*θ*, *ρ*) matrix in the blue (450 nm) spectral range for $d_{\min}^{*}=23\%$ shown in figure 4*b*.

	*θ*_1_ = −5–0°	*θ*_2_ = 0–5°	*θ*_3_ = 5–10°	*θ*_4_ = 10–15°	*θ*_5_ = 15–20°	*θ*_6_ = 20–25°	*θ*_7_ = 25–30°	*θ*_8_ = 30–35°	*θ*_9_ = 35–40°	*θ*_10_ = 40–45°	*θ*_11_ = 45–50°	*θ*_12_ = 50–55°
*ρ*_8_	0.46	4.65	0.71	1.22	6.13	0.47	2.16	3.13	2.75	1.63	7.09	14.96
*ρ*_7_	6.84	7.01	3.71	1.15	10.70	9.85	6.28	8.85	7.92	9.77	13.27	3.98
*ρ*_6_	26.00	20.01	13.07	15.65	12.87	10.82	12.22	7.67	8.92	12.57	11.55	18.51
*ρ*_5_	25.60	19.83	18.37	17.07	20.88	15.65	13.62	10.43	14.52	14.91	10.37	12.09
*ρ*_4_	29.66	18.36	10.42	12.40	9.87	17.58	17.39	15.88	9.53	9.33	7.71	11.19
*ρ*_3_	22.81	19.19	7.60	9.05	7.44	12.48	17.65	15.86	9.85	20.55	13.27	10.29
*ρ*_2_	4.07	6.58	7.33	3.27	7.20	10.12	16.03	14.95	7.98	11.04	9.84	10.45
*ρ*_1_	2.63	3.08	2.68	1.25	6.07	4.866	9.12	7.49	5.57	3.18	1.98	6.90
*ρ*_0_	4.18	1.97	2.66	2.41	3.21	2.12	3.26	5.63	8.91	3.73	4.69	3.52

**Table 5.** Numerical values (%) of the Δ*P*(*θ*, *ρ*) matrix in the blue (450 nm) spectral range for $d_{\mathrm{ave}}^{*}=56\%$ shown in figure 4*b*.

	*θ*_1_ = −5–0°	*θ*_2_ = 0–5°	*θ*_3_ = 5–10°	*θ*_4_ = 10–15°	*θ*_5_ = 15–20°	*θ*_6_ = 20–25°	*θ*_7_ = 25–30°	*θ*_8_ = 30–35°	*θ*_9_ = 35–40°	*θ*_10_ = 40–45°	*θ*_11_ = 45–50°	*θ*_12_ = 50–55°
*ρ*_8_	0.0597	0.1204	0.0506	0.0729	0.0700	0.0746	0.1226	0.0688	0.0554	0.0500	0.0856	0.0513
*ρ*_7_	0.0600	0.0381	0.0473	0.0376	0.1199	0.2273	0.1082	0.2035	0.1325	0.1540	0.1660	0.0392
*ρ*_6_	1.4282	0.4922	0.0736	0.4471	0.1367	0.3437	0.1912	0.1696	0.2246	0.5242	0.3197	0.5472
*ρ*_5_	6.0898	2.4652	0.0492	0.0773	0.3000	0.1133	0.2028	0.1513	0.4601	0.5565	0.9953	0.2599
*ρ*_4_	14.057	3.8582	1.8149	0.9965	0.1290	0.3830	0.3181	0.4286	0.1642	0.1898	0.2064	0.4319
*ρ*_3_	11.902	8.7061	4.0724	2.5452	0.1976	0.4637	0.2381	0.2886	0.3158	1.2297	0.3296	0.2045
*ρ*_2_	7.5923	9.9259	6.1184	3.1408	5.2772	2.7204	0.3430	0.4612	0.8336	0.5078	0.3720	0.1434
*ρ*_1_	9.4660	9.6793	8.6202	6.4879	11.2379	6.0032	4.7326	0.9265	0.7885	2.3646	1.4952	0.3427
*ρ*_0_	10.2622	7.7071	7.4711	8.4811	7.93141	3.7781	4.1875	6.5901	0.2668	0.0396	0.1969	0.0774

**Table 6.** Numerical values (%) of the Δ*P*(*θ*, *ρ*) matrix in the blue (450 nm) spectral range for $d_{\max}^{*}=87\%$ shown in figure 4*b*.

	*θ*_1_ = −5–0°	*θ*_2_ = 0–5°	*θ*_3_ = 5–10°	*θ*_4_ = 10–15°	*θ*_5_ = 15–20°	*θ*_6_ = 20–25°	*θ*_7_ = 25–30°	*θ*_8_ = 30–35°	*θ*_9_ = 35–40°	*θ*_10_ = 40–45°	*θ*_11_ = 45–50°	*θ*_12_ = 50–55°
*ρ*_8_	0.0036	0.0145	0.0040	0.0057	0.0101	0.0012	0.0129	0.0093	0.0038	0.0026	0.0069	0.0028
*ρ*_7_	0.0060	0.0050	0.0039	0.0024	0.0136	0.0212	0.0058	0.0242	0.0130	0.0064	0.0177	0.0011
*ρ*_6_	0.0065	0.0244	0.0143	0.0101	0.0156	0.0265	0.0278	0.0088	0.0121	0.0624	0.0517	0.0295
*ρ*_5_	0.0095	0.0102	0.0104	0.0039	0.0047	0.0160	0.0143	0.0198	0.0248	0.0392	0.1011	0.0390
*ρ*_4_	0.0222	0.0128	0.0045	0.0069	0.0036	0.0243	0.0320	0.0412	0.0195	0.0179	0.0403	0.0745
*ρ*_3_	0.0165	0.0108	0.0080	0.0261	0.0150	0.0152	0.0188	0.0107	0.0379	0.0518	0.0391	0.0426
*ρ*_2_	0.0207	0.0252	0.0201	0.0061	0.0630	0.0138	0.0137	0.0132	0.0040	0.0297	0.0301	0.0234
*ρ*_1_	0.0393	0.0223	0.0186	0.0055	0.0357	0.0232	0.0144	0.0287	0.0017	0.0134	0.0135	0.0265
*ρ*_0_	0.1315	0.2681	0.0293	0.0324	0.0097	0.0034	0.0194	0.0731	0.0103	0.0021	0.0371	0.0034

In the case of a most sensitive person with a perception threshold $d_{\min}^{*}=23\%$, <*P*> has relatively large values (<*P*> ≤ 76.99%) for *ρ* ≤ 75%, especially at solar elevations *θ* ≤ 35° (figure 4*a*, table 1). Therefore, in many such meteorological situations this person is able to perceive Haidinger's brushes and to guess the position of the sun occluded by clouds in relatively large sky regions. For an average person with a perception threshold $d_{\mathrm{ave}}^{*}=56\%$ of Haidinger's brushes, <*P*> has the largest values (<*P*> ≤ 28.14%) for completely or nearly clear skies with *ρ* ≤ 25% and lower sun elevations *θ* ≤ 15° (figure 4*a*, table 2). A person having low polarization sensitivity (e.g. $d_{\max}^{*}=87\%$) can only detect Haidinger's brushes under very few meteorological conditions (figure 4*a*, table 3; <*P*> ≤ 0.1375%, being practically zero) and would, therefore, be an unlikely choice for a Viking navigator.

The most sensitive persons $\left(d_{\min}^{*}=23\%\right)$ possess the widest range of the standard deviation (1.97% ≤ Δ*P* ≤ 29.66%) for medium per cents of cloud cover *ρ* ≤ 75% and solar elevations not larger than 35° (figure 4*b*, table 4). Persons with an average sensitivity $\left(d_{\mathrm{ave}}^{*}=56\%\right)$ have small standard deviations (3.14% ≤ Δ*P* ≤ 10.26%) for smaller per cents of cloud cover *ρ* ≤ 25% and lower solar elevations *θ* ≤ 15° (figure 4*b*, table 5). For the least polarization-sensitive persons (with $d_{\max}^{*}=87\%$) the standard deviations of <*P*> are practically zero (Δ*P* ≤ 0.2681%; figure 4*b*, table 6). The larger the standard deviation Δ*P* at a given meteorological situation (*θ*–*ρ* cell), the smaller is the reliability of the navigation method based on Haidinger's brushes under that sky condition.

## 4. Discussion

In this work we determined the proportion *P* of the sky for which the degree *d* of sky polarization is larger than the perception threshold d* (=23, 56, 87%) of Haidinger's brushes at a given meteorological situation characterized by the per cent cloud cover *ρ* and sun elevation *θ*. The mean <*P*> of *P* (averaged for 12 different skies possessing very similar values of *ρ* and *θ*) is a good measure of the appropriateness of a given sky for Viking navigation with Haidinger's brushes. <*P*> and its standard deviation Δ*P* were determined for all cells of the (*θ*, *ρ*) matrices (figure 4, tables 1–6) with the use of the patterns of the degree of polarization *d* measured with full-sky imaging polarimetry in the blue part of the spectrum. Although we measured the sky polarization for 1296 meteorological situations with different solar elevations *θ* and cloudinesses *ρ* in Hungary at latitude 47°15'29.83^″^ N, this might not affect considerably the sky polarizaton, because for a given *ρ*, celestial polarization depends predominantly on *θ*, and we restricted our measurements to solar elevations –5° ≤ *θ* ≤ +55° occurring at 61° latitude, where the main Viking sailing route ran. The range 0 okta ≤ *ρ* ≤ 8 oktas used by us covered the whole range of cloudiness that can also occur at 61° latitude.

In the analysis of the proportion *P* of the sky for which the degree *d* of sky polarization is larger than the perception threshold d* (=23, 56, 87%), the most important spectral range is the blue, in which the human eye perceives Haidinger's brushes with a maximal sensitivity at 460 nm due to absorption of macular pigments [39]. In blue light the Haidinger's brushes become clearer, and it is thought that the yellow colour a person sees under full-spectrum illumination is a psychophysical effect of the eye where yellow is perceived when there is an absence of blue [1,2]. Thus, our study was restricted to this part of the spectrum. We obtained that the mean <*P*> of *P* is large only for the most sensitive-eyed human observers (Viking navigators) having a perception threshold $d_{\min}^{*}=23\%$ of Haidinger's brushes. Viking navigators were probably those that had the highest polarization sensitivity (i.e. having the lowest threshold d*). A diet rich in fish, which is a good source of macular carotenoids, may have contributed to Vikings' general ability to see Haidinger's brushes (S. Temple 2016, personal communication).

We found that the proportion *P* of the sky suitable for humans with a high sensitivity to perceive Haidinger's brushes $\left(d_{\min}^{*}=23\%\right)$ is high under numerous meteorological conditions, particularly when the sun is low on the horizon (*θ *≈ 0°) and when the sky is clear of clouds (*ρ *≈ 0%). When the sun is below the horizon (−5° ≤ *θ* < 0°), and therefore not possible to be seen or accurately located, the mean proportion <*P*> of the sky suitable for sky-polarimetric navigation is between 26.4% and 76.2%, depending on the cloud cover (*ρ* < 75%).

This finding corresponds to the results of Száz *et al*. [38], who also found that regarding the estimation of solar elevation angle with numbers of fists and fingers of outstretched arms in the third step of sky-polarimetric Viking navigation, the most appropriate time for navigation is around sunset and sunrise. The navigation method deduced by Bernáth *et al*. [31] based on the Uunartoq artefact fragment functioning as a twilight board is useful also during twilight and under clear skies, when the sun is close to the horizon (when −8° ≤ *θ* ≤ +10°). Using a twilight board, a shadow-stick and sunstone crystals could have allowed Vikings to navigate during long twilight periods on the basis of polarization patterns of clear skies. Based on our results in this paper, we can conclude that birefringent (e.g. calcite) or dichroic (e.g. tourmaline or cordierite) sunstone crystals could be replaced with Haidinger's brushes in the sky-polarimetric Viking navigation.

In this work, we investigated only the meteorological prerequisites (solar elevation and per cent cloud cover) of sky-polarimetric Viking navigation based on Haidinger's brushes. However, further factors also determine whether this method can really be used for reliable navigation. For example, it is questionable whether the few seconds prior to fading of the Haidinger's brushes when looking at the polarized sky with a fixed head are enough to determine the position of the occluded sun in the sky. However, these few seconds before fading are irrelevant if our head is tilted periodically back and forth while looking at the sky and then we use an object in the peripheral foreground (e.g. our arm) to point in the direction of the yellow Haidinger's brushes. Nevertheless, it would be worth studying the accuracy and reliability of Viking navigation based on Haidinger's brushes under real open air conditions.

Finally, we emphasize that even if a navigator could see the Haidinger's brushes in the sky under certain conditions, only computations of error propagation through the three steps of sky-polarimetric Viking navigation can decide if the method using Haidinger's brushes is accurate enough. Such computations have been presented by Farkas *et al*. [34] and Száz *et al*. [37,38] for this navigation method using dichroic and birefringent sunstone crystals. It is beyond the scope of this paper to carry out such error propagation to determine the practicality of the navigation method based on Haidinger's brushes. This can be an interesting task of future research.

## Data accessibility

There is no supporting data or supplementary information for this paper.
